# Associations among navigational support and health care utilization and costs in patients with advanced cancer: An analysis based on administrative health insurance data

**DOI:** 10.1002/cam4.5574

**Published:** 2023-01-09

**Authors:** Daniel Schindel, Pimrapat Gebert, Johann Frick, Anne Letsch, Ulrike Grittner, Liane Schenk

**Affiliations:** ^1^ Institute of Medical Sociology and Rehabilitation Science, Charité—Universitätsmedizin Berlin, corporate member of Freie Universität Berlin and Humboldt‐Universität zu Berlin Berlin Germany; ^2^ Institute of Biometry and Clinical Epidemiology, Charité ‐ Universitätsmedizin Berlin, corporate member of Freie Universität Berlin and Humboldt‐Universität zu Berlin Berlin Germany; ^3^ Department of Medicine II, Hematology and Oncology University Hospital Schleswig‐Holstein Kiel Germany

**Keywords:** health care costs, oncology nursing, patient navigation, psychosocial support systems, quality of life, routinely collected health data

## Abstract

**Background:**

Fragmented and complex healthcare systems make it difficult to provide continuity of care for patients with advanced cancer near the end of life. Nurse‐based cross‐sectoral navigation support has the potential to increase patients' quality of life. The objective of this paper was to evaluate associations between navigation support and health care utilization, and the associated costs of care.

**Methods:**

The evaluation is based on claims data from 37 statutory health insurance funds. Non‐randomized recruitment of the intervention group (IG) took place between 2018 and 2019 in four German hospitals. The comparison group (CG) was defined ex post. It comprises nonparticipating clients of the involved health insurance funds matched on age, gender, and diagnosis in a 1:4 ratio to the IG. Healthcare resource utilization was compared using incident rate ratios (IRRs) based on negative binomial regression models. Linear mixed models were performed to compare differences in lengths of hospital stays and costs between groups.

**Results:**

A total of 717 patients were included (IG: 149, CG: 568). IG patients showed shorter average lengths of hospital stays (IG: 11 days [95% CI: 10, 13] vs. CG: 15 days [95% CI: 14, 16], *p* < 0.001). In the IG, 21% fewer medications were prescribed and there were on average 15% fewer outpatient doctor contacts per month. Average billed costs in the IG were 23% lower than in the CG (IG: 6754 EUR [95% CI: 5702, 8000] vs. CG: 8816 EUR [95% CI: 8153, 9533], *p* < 0.001).

**Conclusions:**

The intervention was associated with decreased costs mainly as a result of a non‐intended navigation effect. The social care nurses had navigated patients within the hospital early, needs‐oriented and effectively but interpreted their function less cross‐sectorally. Linkage of hospital‐based navigators with the outpatient care sector needs further exploration.

## Graphical abstract

Cross‐sector navigation support by nurses has the potential to improve patients' quality of life. With this work, we were able to show that there are also beneficial associations between navigation support and health care utilization and associated costs of care.

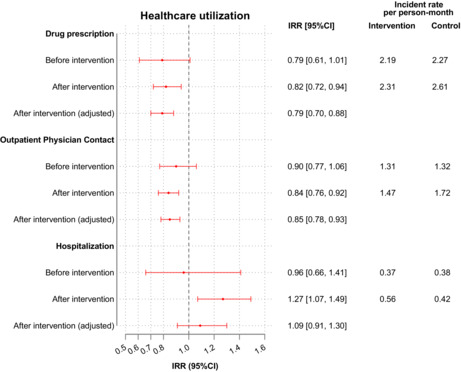

## 1 BACKGROUND

Cancer is currently one of the most frequent causes of early death globally, and will continue to grow in relevance.^1^, ^2^ An estimated 19.3 million people contracted cancer in 2020.^3^ Despite improved survival chances^4^—due to improved diagnosis and treatments–almost 10 million people died of cancer last year. Satisfactory care for patients with advanced cancer near the end of life is a still‐unsolved challenge. In this context, inadequate or excessive care are discussed (“overuse,” “aggressive treatment,” “poor‐quality care”).^5^, ^6^ For patients^7^ and caregiving relatives,^8^ this increases the suffering that already arises directly and indirectly from the diagnosis itself, but also the total economic burden for society.^9^ Unsatisfactory care also frequently contradicts the often‐expressed desire to die at home or in a hospice.^10^, ^11^ Early palliative and outpatient cancer care have already been clearly shown to have positive effects on seriously affected patients' quality of life and mortality, and on economic criteria.^12^, ^13^, ^14^, ^15^ As early as the 1990s, for example, Rubenstein called for a rethinking of the patterns of care that had prevailed until then and to test the benefits of care in the hospital, the outpatient treatment department, and the patient's home.^16^

Possible reasons why traditional forms of care continue to dominate may include the difficulty of making clear prognoses, and interface problems. In Germany, for example, specific treatment of advanced tumor stages usually takes place inpatient in specialist oncological departments. Specialized palliative care is often still provided in inpatient settings, but also in home care or hospices. The division into different care sectors such as inpatient and outpatient care that has occurred over time mirrors increasing functional specializations, and has resulted in a fragmented, often confusing, healthcare system.^17^, ^18^ Patients' vulnerable health situation, in which they are confronted with numerous existential psychosocial issues in addition to the burden of physical symptoms, is made still more difficult by the necessary orientation in complex healthcare systems.^19^, ^20^, ^21^ Reactions to this structural development include interprofessional networking, integrated care models, and navigation approaches. Some navigation issues are still unanswered, including where patient navigators or case managers should be located, what qualifications they require, which (care) tasks they should take on, when they should be deployed, and what their targets should be.^22^ Previous types of implementation range, for instance, from continuous contact persons^23^ to organized support for peers and supervised lay caregivers^24^, ^25^ or experienced nurse navigators.^26^ Some supportive navigation approaches started at early stages of the disease; others are only available in the context of inpatient treatment, or only offer short‐term care.^26^, ^27^

Nurse navigators are in a good position to closely monitor patients' situations using standardized assessment tools and to offer preference‐oriented support, due to their early patient contact and professional qualifications.^25^, ^26^ Alongside coordinated support on psycho‐oncological, social, legal, and coordination issues, a special feature of our intervention is continuous cross‐sector monitoring of patients, including picking up comparable previous care approaches and developing them further.^28^, ^29^

One aim of the intervention was to effectively coordinate treatments and support across sectoral boundaries, thus achieving economic effects previously only reported in a few intervention studies.^30^, ^31^ Previous evaluations of innovative navigation approaches for oncological patients rarely include economic evaluation, often focusing on their effectiveness in relation to defined care outcomes and patient‐reported outcomes (PRO).^31^, ^32^ Bernado et al.'s systematic review included a range of cancer types and reported positive economic outcomes for the navigation programs observed in the majority of cases; the cost‐effectiveness was seen, for example, in reduced costs for medication, less frequent hospital stays, and fewer demands on emergency or intensive care.^31^ Gervés‐Pinquie et al.'s systematic overview evaluates relevant navigation programs specifically for colorectal cancer, including cost effectiveness.^33^ Their study describes both direct and indirect program costs, showing very high costs for the implementation of navigation programs. As a result, they are only cost‐effective for patients with very advanced disease.^33^

In our study, social care nurses (SCN) acted as patient advisors and facilitators, one task being to shape the interface between inpatient and outpatient care more efficiently. Initial analyses of the impact of SCN input showed clinically relevant improvements in quality of life after 6 months and significant improvements in cognitive performance and reduction of symptom burden in five out of nine sub‐scales of the EORTC QLQ‐C30 (tiredness, nausea and vomiting, dyspnea, sleeplessness, appetite loss).^34^

The primary objective of the analyses in this paper is to examine to what extent SCN input moderates advanced cancer patients' utilization behavior in relation to inpatient and outpatient care. The secondary objective is to evaluate how the intervention is associated with the costs of care.

## 2 METHODS

### 2.1 Intervention

The aim of the intervention was to improve patient‐reported quality of life by symptom and preference screening and navigation with social care nurse.^28^ In particular, one objective was to achieve a shift from inpatient to outpatient care, which is linked to improved quality of life and reduction of total costs compared to standard treatment.

Orientated on Kelly et al., the social care nurse navigator could be described as follows.^22^ The nurses were employed by the participating hospitals. They worked in regular shifts on oncological wards and were granted a fixed hourly quota of time off for study on the side. Specially trained navigators are already used in various areas as described at the background section. This intervention adds the cross‐sectoral and continuous care by one and the same nurse over a longer period of one year, irrespective of whether the patients receive outpatient or inpatient care during this period or are temporarily free of therapy. Patients were actively contacted by their personal social care nurse at least once a month by telephone, email, or in a face‐to‐face meeting. The monthly recorded QoL questionnaires (EORTC QLQ‐C30^35^) were used both to assess patient needs and identify gaps in care and for evaluation of the study. The social care nurses' key function was to navigate to and coordinate medical, psychosocial, and palliative support services (e.g., contact to inpatient and outpatient therapists, support groups, early palliative care) and to reduce barriers to receiving timely services. Additional function was to educate patients about the healthcare system. Social care nurses had a professional background. The prerequisite for further training as a social care nurse was a nursing training, occupational training as social worker, or a degree in social pedagogy. The majority of the six social care nurses had additional training in psycho‐oncology. The three‐week training of the SCN is essentially based on the training curriculum for oncologists of the Saxon Cancer Society.^29^

### 2.2 Patients

Non‐randomized recruitment of the intervention group took place between February 1, 2018, and February 1, 2019, in four German hospitals. The group included patients with advanced cancer diagnoses, operationalized by a combination of defined cancer diagnoses and operation and procedure codes: leukemia (ICD‐10 GM code (International Statistical Classification of Diseases and Related Health Problems German Modification (ICD GM)): C91‐C92), lymphoma (ICD‐10 GM code C82‐C86), metastasized colorectal cancer (ICD‐10 GM code C18‐C20, C77‐C79), malignant neoplasm of pancreas (C25), malignant neoplasm of bronchus and lung (C34), multiple myeloma and malignant plasma cell neoplasms (C90), metastasized malignant neoplasm of breast (C50 (+C77‐C79)), metastasized malignant neoplasm of ovary (C56 (+C77‐C79)), metastasized malignant neoplasm of cervix uteri (C53 (+C77‐C79)), metastasized malignant neoplasm of corpus uteri (C54 (+C77‐C79)), malignant neoplasm of stomach (C16), malignant neoplasm of esophagus (C15), metastasized malignant neoplasms of lip, oral cavity, and pharynx (C00–C14 (+C77‐C79)), metastasized malignant neoplasm of prostate (C61 (+C77‐C79)), metastasized malignant neoplasm of thyroid gland (C73 (+C77‐C79)), metastasized melanoma and other malignant neoplasms of skin (C43–C44 (+C77‐C79)) in combination with the following operation and procedures codes (OPS‐Codes): surgical operation on the digestive tract (5‐42–5‐54), surgical operation on the lymphatic tissues (5‐402–5‐404; 5‐406), radiotherapy, nuclear medicine therapy and pain management (8‐52, 8‐53, 8‐91), multimodal pain treatment, cytotoxic chemotherapy, complex treatment (8‐541–8‐544; 8‐546; 8‐918; 8‐982; 8‐98e). All patients included in the study were member in one of the 37 statutory company health insurance funds (BKK). These insurance funds have a common historical background. As company health insurance funds they exclusively insure employees of a particular industrial company or group. Thus, they have a strong industrial connection. The implications of health insurance fund affiliation was discussed in detail in a previous publication.^36^ All patients were at least 18 years old. On the participating wards, each eligible patient was approached by the study team for participation. Additional details of the original study have been comprehensively described.^28^ The project was approved by the ethics committees at Charité – Universitätsmedizin Berlin (EA2/192/17) and the Medical Association of North Rhine (2017429). Intervention group patients were enrolled in the study after providing written informed consent. The processing of the anonymized data of the comparison group was carried out with approval by the federal supervisory authority of the health insurance funds (Federal Office for Social Security). Approval was granted upon request in accordance with § 75 Social Security Code 10 (Transmission of Social Data for Research and Planning).

### 2.3 Data collection

The analysis is based on claims data from 37 statutory company health insurance funds (BKK). Individual claims data in the intervention year and the year previous to entering the study were registered for patients in the intervention group. The comparison group was defined ex post. It was taken among persons insured by the participating health insurance funds, randomly and stratified by age, gender and diagnosis in accordance with the distribution in the intervention group. To increase the test's power, a group relationship of 1:4 in favor of the comparison patients was selected.^37^ The inclusion date for patients in the comparison group was based on the date of hospital admission in 2018 with a diagnosis and operation relevant to the study. Where no suitable ICD codes were available, previous diagnoses from before the start of the study were taken.

### 2.4 Claims data content (variables)

The data are based on billed hospital services [ICD‐10 GM diagnoses, type of hospital care (inpatient), length of stay in days, cost in euros], outpatient services by statutory health insurance physicians [type of service, cost in euros], medication costs [Anatomical Therapeutic Chemical Classification codes (ATC codes), cost in euros], and health services and medical sundries [type of service, cost in euros]. Additionally, sociodemographic patient data was also recorded age [in years], gender [male, female], nationality [German, other], care level [scale 1‐5 (e.g., care level 5 equals the most severe impairment of independence with special requirements for nursing care)]. The date of death was also included for patients who died. In addition, the age‐adjusted Charlson index for each person was determined from the diagnoses to evaluate the individual comorbidity burden.^38^

### 2.5 Economic measurement parameters

Utilization of care services (objective 1) was evaluated based on the average number of outpatient medication prescriptions per month, average number of physicians involved in outpatient care per month, average number of inpatient hospital stays per month, average lengths of stay (in days) per patient. Direct care costs (objective 2) comprise costs of outpatient physician care, inpatient hospital care, medication prescriptions (outpatient medication prescriptions only), health services and medical sundries, and—for the intervention group—intervention program costs (training costs, patient care). Private co‐payments by patients were not included. Due to varied observation periods, total costs during the observation period were averaged, so the costs presented can be interpreted as monthly costs.

### 2.6 Statistical analyses

Baseline characteristics are presented using absolute and relative frequencies for categorical variables. Continuous variables are described using means, standard deviations (SD), medians, and interquartile ranges (IQR). Differences in patient characteristics between groups were analyzed using chi‐square tests for categorical variables and independent *t*‐tests or Mann–Whitney *U*‐tests for continuous variables. Furthermore, effect sizes in the form of standardized mean differences (SMDs) were calculated to check the balance of baseline characteristics between intervention and comparison group in this non‐randomized setting. Values of SMD < ±0.1 are considered an adequate balance between the groups.^39^

Person‐time was calculated from date of enrolment and end of observation (12 months after enrolment, or date of death). Incidence rate (IR) per person‐year was presented for each utilization of healthcare services as healthcare resource utilization (HRU). The HRU outcomes were compared between groups using negative binomial regression models and the results presented as unadjusted and adjusted incidence rate ratios (IRRs) with 95% confidence intervals (CIs). We tested for overdispersion by comparing Poisson regression models and negative binomial regression models using the likelihood‐ratio test. Our data showed overdispersion, thus the negative binomial regression models offer a better fit to our data compared to the Poisson regression models.

Due to the typical right‐skewed distribution of length of stays (LOS) and cost data (Cost), we used log‐transformed LOS (ln‐LOS) and costs (ln‐Cost) for the analysis. The estimated ln‐LOS and ln‐Cost results were retransformed to the original LOS and Cost by exponential the estimated values, which is known as the geometric mean (GM). Before the re‐transformation, homoscedasticity has been checked for a stabilized variance from the estimated regression model^40^, ^41^ by using White's test and the estimated retransformed LOS and costs are adjusted by the Duan's smearing factor^40^, ^42^ when the error term is not normally distributed. Linear regression models were performed to analyze differences of ln‐LOS and ln‐Cost between groups and 95% CI was estimated by robust method. Sensitivity analysis was performed for LOS and cost by using generalized linear models (GLM) using log‐link with a gamma distributional family (Tables S1 and S2). We explored overfitting by comparing adjusted *R* ^2^ between linear regression models and GLM models. The *R* ^2^ of a transformed model are 0.077 and 0.193 for LOS and total cost and the *R* ^2^ of GLM models are 0.045 and 0.184, respectively. Therefore, the log transformation of LOS and costs using linear regression model is not overfit, although there was a slightly better *R* ^2^ when applying a log transformation.

All statistical analyses were performed using Stata IC15 (StataCorp, 2017). Significance was considered at the level of 0.05 without adjustment for multiple testing as the analyses were done in an explorative framework. Interpretation of results is based on effect sizes and estimates and 95% CI. Adjustments for all models were made for age, gender, diagnosis, ACCI (age‐adjusted Charlson comorbidity index), and level of care.

## 3 RESULTS

### 3.1 Study sample and baseline characteristics

**FIGURE 1:**
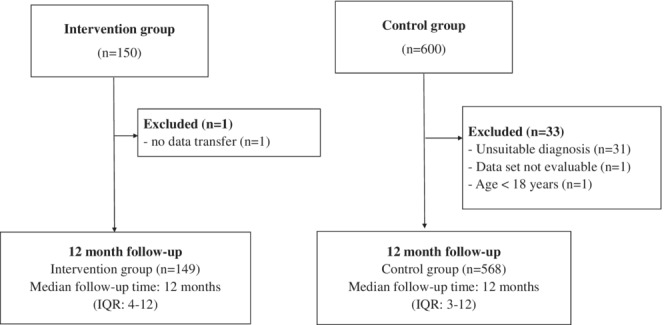
Flow chart for the statutory health insurance data analysis. IQR, interquartile range.

The sample analyzed included *n* = 717 people (Table 1), comprising *n* = 149 people who experienced the intervention (intervention group) and *n* = 568 with no intervention (comparison group). Of the 750 people initially aimed for, *n* = 1 person in the intervention group could not be included due to lack of data. A total of *n* = 33 people in the comparison group could not be included due to unsuitable diagnoses (*n* = 31), lack of data (*n* = 1), or being too young (*n* = 1) (Figure 1).

**TABLE 1:** Baseline characteristics of intervention and comparison group

Characteristics	Total		Intervention group		Comparison group		SMD	*p*‐value
Cases, *n*	717		149		568			
Age (years)								
Mean (SD) [min, max]	66 (14)	[18, 96]	66 (13)	[24, 85]	66 (14)	[18, 96]	−0.014	0.880
<50	71	(9.9%)	12	(8.1%)	59	(10.4%)		
50–59	119	(16.6%)	30	(20.1%)	89	(15.7%)		
60–69	209	(29.2%)	46	(30.9%)	163	(28.7%)		
70–79	223	(31.1%)	41	(27.5%)	182	(32.0%)		
≥80	95	(13.2%)	20	(13.4%)	75	(13.2%)		
Gender, *n* (%)								
Male	415	(57.9%)	84	(56.4%)	331	(58.3%)	0.038	0.676
Female	302	(42.1%)	65	(43.5%)	237	(41.7%)		
Diagnosis, *n* (%)								
Acute Leukemia	55	(7.7%)	19	(12.8%)	36	(6.3%)	−0.069	0.164
Aggressive lymphoma	90	(12.6%)	19	(12.8%)	71	(12.5%)		
Pancreatic cancer	89	(12.4%)	18	(12.1%)	71	(12.5%)		
Lung cancer	177	(24.7%)	31	(20.8%)	146	(25.7%)		
Metastasized colorectal cancer	172	(24.0%)	33	(22.2%)	139	(24.5%)		
Plasmacytoma and malignant neoplasms	31	(4.3%)	5	(3.4%)	26	(4,6%)		
Metastasized mastocarcinoma	23	(3.2%)	3	(2.0%)	18	(3.2%)		
Other	80	(11.2%)	21	(14.1%)	60	(10.6%)		
Year of diagnosis								
Before 2018	302	(42.1%)	63	(42.3%)	239	(42.1%)	−0.004	0.964
Since/after 2018	415	(57.9%)	86	(57.7%)	329	(57.9%)		
Nationality								
German	715	(99.7%)	148	(99.3%)	567	(99.8%)	0.076	0.308
Other nationality	2	(0.3%)	1	(0.7%)	1	(0.2%)		
ACCI score								
Mean (SD)	7.4	(4.2)	7.9	(4.2)	7.3	(4.2)	0.139	0.134
0–1	39	(5.4%)	11	(7.4%)	28	(4.9%)		
2–3	140	(19.5%)	20	(13.4%)	120	(21.1%)		
4–5	109	(15.2%)	18	(12.1%)	91	(16.0%)		
>5	429	(59.8%)	100	(67.1%)	329	(57.9%)		
Care level								
Care level 0	516	(72.0%)	101	(67.8%)	415	(73.1%)	−0.002	0.453
Care level 1	14	(2.0%)	7	(4.7%)	7	(1.2%)		
Care level 2	78	(10.9%)	20	(13.4%)	58	(10.2%)		
Care level 3	56	(7.8%)	15	(10.1%)	41	(7.2%)		
Care level 4	43	(6.0%)	6	(4.0%)	37	(6.5%)		
Care level 5	10	(1.4%)	0	(0.0%)	10	(1.8%)		

Abbreviations: ACCI, age‐adjusted Charlson Comorbidity Index; SMD, Standardized mean difference, Care level 5 = Most severe impairment of independence with special requirements for nursing care.

Due to stratified sampling, there were no substantial group differences between intervention and comparison groups in most characteristics when observation started or in the 12 months prior to the study start, respectively (Table 1). The average age of both groups was 66 years (SD 13 or 14 years). Men were in the majority in both groups. The proportion of acute leukemia was twice as high in the intervention group as in the comparison group (IG = 12.8 vs. CG = 6.3%). The median age‐adjusted Charlson Comorbidity Index (ACCI) score was one point higher in the intervention group (9 vs. 8 points).

### 3.2 Utilization of care services

**FIGURE 2:**
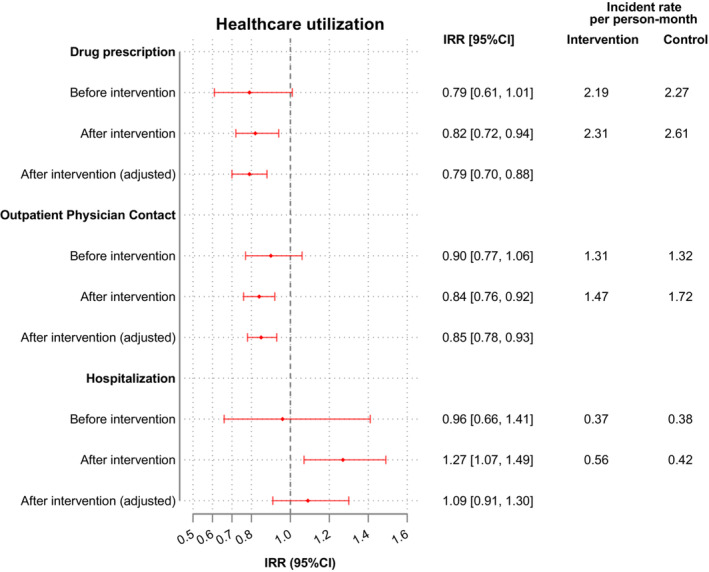
Utilization of care services between intervention group and comparison group. CI, confidence interval, IRR, incidence rate ratio. IRRs were estimated using negative binomial regression models. The IRR was calculated using the comparison group as reference group. Adjusted for age, gender, diagnosis, ACCI score, care level, IRR of utilization of healthcare before begin of intervention.

Before implementation of the intervention, the two groups did not differ in the number of physicians involved in their outpatient care. However, as the study progressed there emerged a difference and was still observable after adjusting for covariates (e.g., age, sex, diagnosis, ACCI score, care level, utilization before OSCAR begin). Patients in the intervention group had an average of 15% fewer outpatient physician contacts per month than patients in the comparison group. (IRR: 0.85 [95% CI: 0.78, 0.93], *p* < 0.001) (Figure 2). Hospital care frequency did not differ between the two groups in conjunction with the intervention. As a result of the regular social care support the intervention group patients were slightly often in hospital than those in the comparison group (IRR: 1.09 [95% CI: 0.91, 1.30], *p* = 0.346) (Figure 2, Table 2).

**TABLE 2:** Incidence rate of utilization of healthcare per person‐month

Utilization of healthcare	Intervention group			Comparison group			Unadjusted^a^		Adjusted^a^ ^,^ ^b^	
	Number of events	Person—month	Incidence rate (per Person—month) (95% CI)	Number of events	Person—month	Incidence rate (per Person—month) (95% CI)	IRR (95% CI)	*p*‐value	IRR (95% CI)	*p*‐value
Observation period (months) – median (IQR)										
Before intervention	12 (12, 12)			12 (12, 12)						
After intervention	12 (4, 12)			12 (3, 12)						
Medication prescriptions										
Before intervention	2807	1279	2.19 (2.11–2.28)	9918	4366	2.27 (2.23–2.32)	0.79 (0.61–1.01)	0.065	—	—
After intervention	3033	1312	2.31 (2.23–2.40)	11,823	4527	2.61 (2.56–2.66)	0.82 (0.72–0.94)	0.003	0.79 (0.70–0.88)	<0.001
Outpatient physician contacts										
Before intervention	2350	1788	1.31 (1.26–1.37)	8863	6707	1.32 (1.29–1.35)	0.90 (0.77–1.06)	0.213	—	—
After intervention	1924	1310	1.47 (1.40–1.54)	7748	4516	1.72 (1.68–1.75)	0.84 (0.76–0.92)	<0.001	0.85 (0.78–0.93)	<0.001
Admissions to hospital										
Before intervention	415	1135	0.37 (0.33–0.40)	1397	3692	0.38 (0.36–0.40)	0.96 (0.66–1.41)	0.847	—	—
After intervention	722	1289	0.56 (0.52–0.60)	1898	4529	0.42 (0.40–0.44)	1.27 (1.07–1.49)	0.005	1.09 (0.91–1.30)	0.346

^a^ Unadjusted and adjusted values were analyzed using negative binomial regression models.

^b^ Adjusted for age, gender, diagnosis, ACCI score (age‐adjusted Charlson Comorbidity Index), care level, IRR utilization of medical care before OSCAR start.

### 3.3 Length of hospital stay

In the 12 months before study inclusion, no differences in length of hospital stay were observed between intervention and comparison groups (Table 3). The average length of inpatient hospital stay was approx. 12 days. During the study, the average length of hospital stay decreased from 12 to 11 days in the intervention group, and increased from 12 to 15 days for the comparison group (Table 3). After the intervention, therefore, the average inpatient hospital stay decreased by 22% (adjusted exp(β) 0.78, [95% CI: 0.68, 0.89], p < 0.001), approximately 4 days shorter in the intervention group than in the comparison group.

**TABLE 3:** Average length of hospital stay per patient before and after the intervention

Average length of stay (days) per patient	Intervention group		Comparison group		exp(β)^a^ (95% CI)			
	Numbers of patients	LOS (day) (95% CI)	Numbers of patients	LOS (day) (95% CI)	Unadjusted	*p*‐value	Adjusted^b^	*p*‐value
Before intervention	118	12 (11, 14)	443	12 (11, 13)	1.00 (0.86, 1.17)	0.956	—	—
After intervention	141	11 (10, 13)	550	15 (14, 16)	0.79 (0.69, 0.90)	0.001	0.78 (0.68, 0.89)	<0.001

*Note*: Before intervention = average values of the year before inclusion in the study. After intervention = average values after intervention.

LOS (day) is a geometric mean, which is the retransformed mean value from transformed data (ln‐LOS). The estimated mean value of ln‐LOS is retransformed to an exponential scale and the retransformed LOS are adjusted by Duan's smearing factor. The assumption of homoscedasticity is valid (*p*‐value = 0.857, White's test).

^a^ Unadjusted and adjusted values were calculated using a linear regression model; the dependent variable “length of stay” was log‐transformed (ln‐LOS). The estimation was carried out based on the potentiation of the coefficient (β) of the log‐transformed length of stay (LOS).

^b^ Adjusted for age, gender, diagnosis, ACCI score, care level.

### 3.4 Frequency of prescriptions

Twelve months before study inclusion, the number of medication prescriptions were the same in both groups (IR: 2.19 vs. 2.27 per person per month) (Figure 2, Table 2). During the study period, incidences increased in both groups, but not to the same extent. In the intervention group, 21% fewer medications were prescribed compared to the comparison group (IRR: 0.79 [95% CI: 0.70, 0.88], *p* < 0.001).

### 3.5 Healthcare costs

Before the study began, no statistically relevant differences were seen between the intervention and comparison groups in relation to billed costs for medication, treatment by outpatient physicians, hospital stays, health services, and medical sundries (Table 4). During the study and as the disease progressed, billed costs increased in both groups. After the intervention, the total billed costs per patient‐month averaged 6,754 EUR in the intervention group, around 2,062 EUR lower than the comparison group, a reduction of 23% (adjusted exp(β): 0.77 [95% CI: 0.63, 0.92], *p* = 0.006). The savings were driven by approx. 22% lower costs for medication, and approx. 37% lower costs for inpatient hospital services (adjusted exp(β): 0.63 [95% CI: 0.49, 0.83], *p* < 0.001). Economic effects in the form of reduced costs for outpatient physician care were also seen. On average, monthly expenses were reduced by 15% (adjusted exp(β): 0.85 [95% CI: 0.67, 1.08], *p* = 0.179) (Table 4).

**TABLE 4:** Average monthly healthcare costs before and after the intervention

	Intervention group			Comparison group			Unadjusted^c^			Adjusted^c^ ^,^ ^d^		
	*n*	Cost (€)	(95% CI)	*n*	Cost (€)	(95% CI)	exp(β)	(95% CI)	*p*‐value	exp(β)	(95% CI)	*p*‐value
Total costs^a^												
Before intervention	149	2871	(2284, 3610)	568	2254	(1965, 2585)	1.27	(0.98, 1.66)	0.075		—	
After intervention	149	6754	(5702, 8000)	568	8816	(8153, 9533)	0.77	(0.62, 0.96)	0.018	0.77	(0.63, 0.92)	0.006
Medication prescriptions												
Before intervention	146	801	(573, 1120)	559	812	(685, 962)	0.99	(0.68, 1.44)	0.943		—	
After intervention	148	1244	(981, 1578)	555	1603	(1400, 1836)	0.76	(0.55, 1.05)	0.092	0.78	(0.59, 1.02)	0.069
Outpatient care												
Before intervention	149	175	(150, 204)	568	179	(163, 196)	0.98	(0.82, 1.17)	0.826		—	
After intervention	144	366	(293, 458)	548	432	(390, 478)	0.75	(0.58, 0.96)	0.023	0.85	(0.67, 1.08)	0.179
In‐patient hospital care												
Before intervention	128	1655	(1316, 2080)	477	1584	(1390, 1806)	1.04	(0.80, 1.36)	0.747		—	
After intervention	147	4148	(3263, 5272)	566	6548	(5899, 7268)	0.70	(0.53, 0.91)	0.008	0.63	(0.49, 0.83)	0.001
Therapeutic devices and remedies												
Before intervention	84	100	(73, 137)	267	103	(86, 123)	0.97	(0.67, 1.39)	0.850		—	
After intervention	105	265	(199, 352)	354	272	(224, 330)	0.86	(0.63, 1.19)	0.370	0.97	(0.69, 1.38)	0.878
OSCAR cost^b^	149	48.72	—	—	—	—						

Abbreviations: Cost (€) is a geometric mean, which is the retransformed mean value from transformed data (ln‐Cost). The estimated mean value of ln‐Cost is retransformed to an exponential scale and the retransformed costs are adjusted by Duan's smearing factor. The assumption of homoscedasticity is valid for all costs (White's test).

^a^ Total costs were summarized of all costs: medication prescriptions, outpatient care, in‐hospital care (including Ambulance, partial in‐patient, and in‐patient care), therapeutic devices and remedies, and OSCAR cost (only intervention group).

^b^ OSCAR costs were estimated from average costs per OSCAR patient of ca. 584.64 € and monthly costs of ca. 48.72€.

^c^ Unadjusted and Adjusted estimates were calculated using linear regression models; the dependent variable ‘Cost (€)’ was log‐transformed (ln‐Cost). The estimation was carried out based on the potentiation of the coefficient (β) of the log‐transformed Cost, where exp(β) = the exponentiated regression coefficients of log‐transformed costs.

^d^ Adjusted for age, sex, diagnosis, ACCI score, level of care, costs before begin of intervention.

The average program costs per patient‐month were 48.70 EUR, including costs for training the SCN and the monthly navigation and counseling talks.

## 4 DISCUSSION

The intended shift from inpatient care to outpatient provision through input from SCNs was not achieved. Nevertheless, a positive economic outcome can be seen from the SCNs' input in navigation for patients with advanced cancer and poor prognosis, resulting from shorter hospital stays and fewer prescriptions for medication, health services, and medical sundries. These effects also coincide with the patients' stated care preferences.

### 4.1 Utilization of care services and lengths of hospital stays

As the disease progressed and the intervention was introduced, changes were observed in the utilization of care services in both patient groups. The assumptions based on prior literature of the association of decreasing care costs due to a decrease in hospitalizations accompanied by an increase in outpatient contacts was not observed.^15^, ^16^

High utilization levels of inpatient care services by patients with advanced disease within the German healthcare system were also previously described by Schneider et al.^43^ However, they report frequencies of between 2 and 3.3 admissions in the final year of life, well below that of the populations observed here. This difference could be due to the wide range of diagnoses included in our study. Equally, Schroeder et al. describe clear differences in hospital admissions frequency depending on the oncological entity.^43^ In contrast, Reeves et al. showed a similar monthly admission probability for oncological patients in the last 3 months before their deaths, based on routine data in Australia.^44^

As described above, there is profound evidence for the effectiveness of patient navigation models. The reduction in average lengths of hospital stay observed by us in comparison to regular care was, however, rarely intended or focused on in previous program evaluations. Bakitas et al. observed no reduction in days in hospital or reduced admissions resulting from their program; however, improved quality of life was reported.^45^ The intervention described here showed both effects.^34^ The average hospital stay we observed in the intervention group was well below that in the available reference studies. Schneider et al. published hospital stays of between 20 and 35 days for patients with similarly severe disease, reporting large differences between the entities.^43^ However, because average lengths of stay in Germany have generally decreased in recent years, Schneider et al.'s findings from 2007 are only comparable to current figures to a very limited extent.^46^

But how to explain the shorter average length of stays and lower utilization of outpatient services compared with the comparison group? The SCNs who worked on cancer wards, gave them easier access to the highly vulnerable and hard‐to‐reach patient population. Faller et al., for example, showed that acceptance for psychological counseling and support services for cancer patients varied according to the setting.^47^ They observed that patients in an outpatient setting utilized services of this type less often than patients in hospital.^47^ The SCN's local links in hospital also had an unintended navigation effect. Most nurses who received the additional SCN qualification were cancer specialists who had worked in hospital for years and were familiar with its structures and networks, as well as having specialist knowledge of suitable treatments. Clearly, the SCNs interpreted their networking function internally rather than as a cross‐sector task, succeeding in navigating the patients within the hospital at an early stage, appropriately to their needs, and effectively.

Finally, inpatient care might have corresponded to the intervention patients' wishes, in a sense as the result of an increasingly trusting relationship to the SCN and the hospital. Care needs may have been discussed more directly and openly.^48^, ^49^ Jacobsen et al. also address this issue in their impressive portrayal of patients who expressed the wish not to be a mere number or one of many in the system. This wish “to be known” is addressed by the stable personal relationship and regular contacts to the SCN.^50^

This aspect is also reflected here in the patient reported outcomes measures such as quality of life. For example, patients in the intervention group reported quicker gains in quality of life and reduced symptom burden due to counseling and navigation by the social care nurses.^34^ This continuous cross‐sector service also closes a significant orientation gap as already described by Schoen et al., for example by helping plan a stay in hospital or clarifying the responsibilities of individual care actors.^21^

### 4.2 Prescriptions

Previous studies or systematic reviews do not describe a steering effect on medication prescriptions by oncological navigation programs. The intervention effect observed here of a reduction in medication prescriptions seems interesting in view of a reduction in polypharmacy and undesirable medication side‐effects, and should be given more attention.^51^, ^52^ A possible explanation for this effect could be the reduced utilization of outpatient physicians' involvement, compared to the comparison group. Prescription medications for home use are exclusively prescribed by outpatient physicians in the outpatient sector in Germany; where fewer visits to outpatient physicians take place, the likelihood of supplementary prescriptions is correspondingly lower.^17^

### 4.3 Healthcare costs

Despite increased utilization of inpatient care, our study's intervention proved cost‐effective. In two systematic reviews, positive cost effects of most of the patient navigation programs they included were confirmed by Bernado et al. for a wide diagnostic spectrum and by Gervès‐Pinquié et al. for patients with colorectal cancer.^31^, ^33^ We should point out that the studies they included are very heterogeneous in terms of the profiles and tasks of the navigators (e.g., from lay patient navigators up to hospital‐based clinical nurse specialists,^33^ from increased rates of screening, and diagnostic resolution up to cancer control navigation^31^). Only two of the studies included in^31^ and one in^33^ reported no return on investment. The mean program costs of 585 EUR per year for the program described here are about average for comparable interventions (reference studies: 275 to 2080 USD).^31^ The cost‐effectiveness achieved here resulted primarily from savings in inpatient care due to shorter stays, and reduced costs for medication and outpatient services. In contrast, other navigation programs amortized via a reduction in hospital admissions, lower utilization of emergency departments and intensive care, and earlier admission of patients into hospices.^30^, ^31^, ^53^ Other navigation programs saved costs due to better adherence, appointment attendance and diagnostic certainty, important instruments in countering the further progress of the disease.^31^, ^54^, ^55^ (Early) palliative care provision was also discussed with the SCNs where necessary. This has beneficial effects on quality of life and survival duration as well as positive economic effects.^12^, ^56^ Finally, cross‐sector navigation programs counter the increasing fragmentation of the health system and can contribute to the more efficient organization of care due to the reduction in the number of care providers involved and unnecessary services.^17^, ^57^

## 5 LIMITATIONS

The comparison group was selected ex post from claims data of 37 company health insurance funds, a randomly selected sample stratified according to three characteristics. Uncertainty remains as to whether these characteristics (age, gender, diagnosis) actually represent a comparable group along with their care and utilization. Because company health insurance funds were originally trade‐specific organizations, and work is related to several health‐relevant features, however, health insurance funds could differ in the social and regional make‐up of their clients. Nor was it possible to check regional and clinics‐related effects. However, we consider that the sample is robust because of its disproportionate size. This is also indicated by a similar distribution of other characteristics such as the ACCI, not included in the baseline stratifying characteristics. Nevertheless, there is a general risk of unobserved differences between the study groups, especially in this highly vulnerable patient group with advanced cancer. We performed a survival analysis between IG and CG based on a Kaplan–Meier estimate. Analysis showed a survival rate of 54% in the IG and 58% in the KG. There was no statistically significant difference in survival rates between the two groups. Therefore, we assume that the groups do not differ in their treatment preferences, for example, that one group prefers palliative care even more and consequently uses fewer services, resulting in lower costs.

Inpatient medication treatments and the related costs could not be reflected in isolation in the data and therefore could not be compared between the intervention and comparison group. However, they are part of the total cost and there are indications that inpatient medication provision for the intervention group was not higher than in the comparison group. To that, analyses based on administrative health insurance data are at risk of residual confounding due to a lack of individuals' subjective overall health and not recording reasons for utilization and non‐utilization of health care services.

Finally, the data were collected for billing purposes, not for research. Not all the information required for comprehensive cost evaluations was available: for example, information about non‐medical and indirect costs.^32^ One point of criticism of health insurances' routine data is the quality of their documentation, which affected the intervention and comparison groups equally.

## 6 CONCLUSION

Overall, SCN input for navigation and counseling for patients with advanced cancer was associated with a positive economic effect, which seems notable in times of growing health expenditure with a simultaneous decrease of paying members in the case of contribution‐financed healthcare systems. The savings were not achieved as intended, by a navigation effect across sectoral boundaries into the outpatient sector. Future research should examine the issue of whether and how the advantages of appointing a navigator in hospital can be linked to positive navigation effects in the outpatient care sector, to take patient preferences into account to an even greater extent. Testing the intervention in a larger study group independent of the health insurance type, but also beyond the cancer diagnoses and severities included so far seems desirable, since the intervention addresses a core, highly topical problem of current health care systems.

## AUTHOR CONTRIBUTIONS

**Daniel Schindel:** Conceptualization (equal); data curation (equal); funding acquisition (equal); project administration (equal); supervision (equal); validation (equal); writing – original draft (lead); writing – review and editing (lead). **Pimrapat Gebert:** Conceptualization (equal); data curation (equal); formal analysis (lead); methodology (equal); software (equal); validation (equal); visualization (equal); writing – original draft (equal). **Johann Frick:** Data curation (equal); project administration (equal); writing – review and editing (equal). **Anne Letsch:** Project administration (equal); supervision (equal); validation (equal); writing – review and editing (equal). **Ulrike Grittner:** Data curation (equal); formal analysis (equal); methodology (equal); supervision (equal); writing – review and editing (equal). **Liane Schenk:** Conceptualization (equal); funding acquisition (lead); project administration (equal); supervision (lead); validation (equal); writing – original draft (equal).

## FUNDING INFORMATION

This work was supported by funding from the Innovation Fund of the German Federal Joint Committee. Grant number 01NVF17016.

## CONFLICT OF INTEREST

The authors have no conflict of interest to declare.

## ETHICS STATEMENT

The project was approved by the Ethics Committee of the Charité–Universitätsmedizin Berlin, (EA2/192/17) and the North Rhine Medical Association (2017429).

## CLINICAL TRIAL REGISTRATION

German Clinical Trials Register (DRKS‐ID: DRKS00013640); registered on December 29, 2017.

## Supporting information


Table S1:

## Data Availability

The data that support the findings of this study are available from the corresponding author (daniel.schindel@charite.de) and institution (medsoz@charite.de) upon reasonable request.
