# Supplementary material for: Associations among navigational support and health care utilization and costs in patients with advanced cancer: An analysis based on administrative health insurance data
Source: Cancer Med. 2023 Jan 9;12(7):8662–75. doi: 10.1002/cam4.5574 (PMC10134282; doi:10.1002/cam4.5574)
Supplement: Supplementary file 1 — Table S1: [file CAM4-12-8662-s001.docx]

**Sensitivity analysis**

**Supporting information Table 1: Average length of hospital stay per patient before and after the intervention**

| Average length of stay (days) per patient | Intervention group | | Control group | | Coefficient^a^ (95%CI^c^) | p-value |
| --- | --- | --- | --- | --- | --- | --- |
|  | Numbers of patients | Estimated LOS  (95%CI) | Numbers of patients | Estimated LOS (95%CI) |  |  |
| Before intervention | 118 | 12  (9, 14) | 443 | 10  (9, 11) | 0.12 (-0.09, 0.33) | 0.253 |
| After intervention | 141 | 11  (10, 13) | 550 | 14  (13, 15) | -0.23^b^ (-0.40, -0.07) | 0.006 |

Before intervention=average values of the year before inclusion in the study. After intervention=average values after intervention.

^a^ Unadjusted and adjusted values were calculated using a generalized linear model with log-link and gamma distributional family; the dependent variable ‘length of stay (day)’. The estimation was carried out based on the potentiation of the coefficient of the length of stay (LOS).
^b^ Adjusted for age, gender, diagnosis, ACCI score, care level.

^c^ 95%CI was based on a normal-based bootstrapping method with 100 replications.

**Supporting information Table 2: Average monthly healthcare costs before and after the intervention**

|  | Intervention group | | | Control group | | | Coefficient^c^ | (95%CI^e^) | p-value |
| --- | --- | --- | --- | --- | --- | --- | --- | --- | --- |
|  | n | Cost (€) | (95%CI) | n | Cost (€) | (95%CI) |  |  |  |
| **Total costs^a^** |  |  |  |  |  |  |  |  |  |
| Before intervention | 149 | 2503 | (2035, 2972) | 568 | 2332 | (2072, 2592) | 0.07 | (-0.15, 0.29) | 0.529 |
| After intervention | 149 | 7913 | (6427, 9399) | 568 | 9667 | (8439, 10895) | -0.20 | (-0.40, -0.004) | 0.045^d^ |
| **Medication prescriptions** |  |  |  |  |  |  |  |  |  |
| Before intervention | 146 | 879 | (568, 1190) | 559 | 791 | (652, 930) | 0.10 | (-0.27, 0.48) | 0.584 |
| After intervention | 148 | 1427 | (1084, 1770) | 555 | 2012 | (1716, 2307) | -0.34 | (-0.62, -0.07) | 0.014^d^ |
| **Outpatient care** |  |  |  |  |  |  |  |  |  |
| Before intervention | 149 | 180 | (128, 232) | 568 | 177 | (159, 194) | 0.02 | (-0.30, 0.34) | 0.903 |
| After intervention | 144 | 499 | (328, 671) | 548 | 485 | (385, 585) | 0.03 | (-0.26, 0.32) | 0.843^d^ |
| **In-patient hospital care** |  |  |  |  |  |  |  |  |  |
| Before intervention | 128 | 1639 | (1208, 2070) | 477 | 1584 | (1357, 1811) | 0.03 | (-0.27, 0.33) | 0.823 |
| After intervention | 147 | 5812 | (3850, 7774) | 566 | 7472 | (6169, 8775) | -0.25 | (-0.57, 0.07) | 0.123^d^ |
| **Therapeutic devices and remedies** |  |  |  |  |  |  |  |  |  |
| Before intervention | 84 | 101 | (67, 134) | 267 | 103 | (82, 125) | -0.03 | (-0.40, 0.35) | 0.893 |
| After intervention | 105 | 265 | (167, 362) | 354 | 334 | (230, 439) | -0.23 | (-0.51, 0.04) | 0.096^d^ |
| **OSCAR cost^b^** | 149 | 48.72 | - |  |  |  |  |  |  |

^a^ Total costs were summarized of all costs: medication prescriptions, outpatient care, in-hospital care (including Ambulance, partial in-patient, and in-patient care), therapeutic devices and remedies, and OSCAR cost (only intervention group)

^b^ OSCAR costs were estimated from average costs per OSCAR patient of ca. 584.64 € and monthly costs of ca. 48.72€.

^c^ Unadjusted and adjusted values were calculated using a generalized linear model with log-link and gamma distributional family; the dependent variable ‘length of stay (day)’. The estimation was carried out based on the potentiation of the coefficient of the length of stay (LOS).

^d^ Adjusted for age, sex, diagnosis, ACCI score, level of care, costs before begin of intervention.

^e^ 95%CI was based on a normal-based bootstrapping method with 100 replications.
